# HMG-Coenzyme A Reductase as a Drug Target for the Prevention of Ankylosing Spondylitis

**DOI:** 10.3389/fcell.2021.731072

**Published:** 2021-10-06

**Authors:** Zhenyu Zhong, Xiaojie Feng, Guannan Su, Liping Du, Weiting Liao, Shengyun Liu, Fuzhen Li, Xianbo Zuo, Peizeng Yang

**Affiliations:** ^1^The First Affiliated Hospital of Chongqing Medical University, Chongqing Key Laboratory of Ophthalmology and Chongqing Eye Institute, Chongqing, China; ^2^The First Affiliated Hospital of Zhengzhou University, Zhengzhou, China; ^3^The First Affiliated Hospital of Anhui Medical University, Hefei, China

**Keywords:** HMGCR, ankylosing spondylitis, PCSK9, NPC1L1, genetic variant

## Abstract

Statins are an inhibitor of 3-hydroxy-3-methylglutaryl coenzyme A reductase (HMGCR). Growing evidence indicates that statins may have an anti-inflammatory effect. Whether genetically proxied HMGCR inhibition can reduce the risk of ankylosing spondylitis is unknown. We constructed an HMGCR genetic score comprising nearly randomly inherited variants significantly associated with LDL cholesterol levels within ± 100 kb from HMGCR to proxy for inhibition of HMGCR. We also constructed PCSK9 and NPC1L1 scores as well as the LDL polygenetic score to proxy for the inhibition of these drug targets as well as serum LDL cholesterol levels, respectively. We then compared the associations of these genetic scores with the risk of ankylosing spondylitis. Of 33,998 participants in the primary cohort, 12,596 individuals had been diagnosed with ankylosing spondylitis. Genetically proxied inhibition of HMGCR scaled to per mmol/L decrease in LDL cholesterol levels by the HMGCR score was associated with a lower risk of ankylosing spondylitis (OR, 0.57; 95% CI, 0.38–0.85; *P* value = 5.7 × 10^–3^). No significant association with ankylosing spondylitis was observed for the PCSK9 score (OR, 0.89; 95% CI, 0.68–1.16) and the NPC1L1 score (OR, 1.50; 95% CI, 0.39–5.77). For the LDL score, genetically determined per mmol/L decrease in LDL cholesterol levels led to a reduced risk of ankylosing spondylitis (OR, 0.64; 95% CI, 0.43–0.94), with significant heterogeneity and pleiotropy in the estimate. Exploratory analyses showed that genetically proxied inhibition of HMGCR appeared to have a similar effect to long-term statin therapy in modifying the risk of coronary artery disease and type 2 diabetes, suggesting that the HMGCR score might be a reliable model to assess the effect of statin. Genetically proxied inhibition of HMGCR was associated with a decreased risk of ankylosing spondylitis. This mechanism-based estimate was in line with existing observations suggesting the clinical benefits of statin therapy for ankylosing spondylitis.

## Introduction

Statins are a competitive inhibitor of 3-hydroxy-3-methylglutaryl coenzyme A (HMG-CoA) reductase, the rate-limiting enzyme for cholesterol synthesis. Intervention with statins can reduce serum low-density lipoprotein (LDL) cholesterol levels and is considered as the primary prevention for cardiovascular events (U. S. Preventive Services Task Force, 2016). It is increasingly recognized that inflammation is fundamental to atherogenesis (Wolf and Ley, 2019). Clinical observations and experimental studies indicate that statins may exert their cardiovascular benefits by producing lipid-lowering effects simultaneously along with an anti-inflammatory potential (Antonopoulos et al., 2012). However, causal evidence supporting an independent anti-inflammatory effect of statin therapy has not been well established, which limits its application in inflammatory disease.

Ankylosing spondylitis is a chronic, progressive and inflammatory disease which can affect the sacroiliac joints, spine, peripheral joints, and even extra-articular organs such as the eye. Persistent progression of the disease may lead to disability, comorbidity such as uveitis and visual loss, or even mortality related to excess cardiovascular risk (Haroon et al., 2015; Yang et al., 2018). Therapeutic options are currently restricted to non-steroidal anti-inflammatory drugs (NSAIDs) and immunomodulatory agents for the treatment of signs and symptoms (Dougados and Baeten, 2011). An unmet medical need remains for novel therapies to prevent disease progression and disability. Results from two pilot trials have suggested that short-term statin therapy can lead to the improvement of disease activity and reductions of inflammatory biomarkers in ankylosing spondylitis (van Denderen et al., 2006; Garg et al., 2015). However, whether there is a measurable long-term benefit for statin treatment in ankylosing spondylitis remains unclear.

To provide a biological interpretation for clinical observations as well as a future direction for larger trials, we performed a drug target validation to examine whether genetic variation of HMG-CoA reductase (HMGCR) is associated with the risk to develop ankylosing spondylitis. The functional variants in the gene that encodes HMG-CoA reductase can act as a proxy for pharmacological intervention with statins by inhibition of HMG-CoA reductase (Würtz et al., 2016). Because genetic variants cause a lifelong stable exposure, evaluation of the association of such variants with ankylosing spondylitis may help to provide a robust estimate for the long-term effect of statin therapy on this disease (Walker et al., 2017). A similar approach has successfully been applied in cardiovascular disease, providing estimates on the long-term efficacy and safety outcomes of statins as well as other lipid-lowering drugs (Ference et al., 2015, 2016; Swerdlow et al., 2015). Using the same methodology we also examined the associations of other lipid-lowering drug targets, including PCSK9 (proprotein convertase subtilisin/kexin type 9; target of PCSK9 inhibitors) and NPC1L1 (NPC1 like intracellular cholesterol transporter 1; target of ezetimibe), and LDL cholesterol-lowering effects with the risk of ankylosing spondylitis.

## Materials and Methods

### Study Cohorts and Data Sources

The primary cohort of this study included 33,998 participants enrolled from five genome-wide association study (GWAS) datasets on ankylosing spondylitis, involving individual-level data from 4,913 participants of Chinese descent and summary-level data from 2,012 participants of a Turkish cohort, 1309 of an Iranian cohort and a collection consisted of 22,647 individuals of European ancestry and 3,117 of East Asian ancestry enrolled in International Genetics of Ankylosing Spondylitis Consortium (Table 1) (International Genetics of Ankylosing Spondylitis Consortium et al., 2013; Li et al., 2019). The 1984 modified New York criteria were used to determine the outcome of ankylosing spondylitis in all the above studies (Linden et al., 1984). We also used the datasets of GWAS meta-analyses on coronary artery disease involving 86,995 individuals and on type II diabetes involving 659,316 individuals both of European ancestry in exploratory analyses (Schunkert et al., 2011; Xue et al., 2018). We obtained summary datasets reported by the Global Lipids Genetics Consortium on LDL cholesterol levels based on 188,578 participants for the exposure assessment in this study (Willer et al., 2013). The covariate and multivariate analyses in our study additionally included four GWAS datasets involving a total of 2,509,298 individuals, that included data on one of the following traits: 25-hydroxyvitamin D levels (Jiang et al., 2018), smoking initiation (Liu et al., 2019; Matoba et al., 2019), drinking per week (Liu et al., 2019), and overall physical activity time (Doherty et al., 2018) (Supplementary Table 1). Additional details for each included study are provided in the Supplementary Appendix. All contributing studies were approved by their local research ethics authorities, and written informed consent was obtained from all participants.

**TABLE 1:** Summary of ankylosing spondylitis genome-wide association studies.

GWAS dataset	No. of cases	No. of controls	Genotyping platform
IGAS European cohort	9,069	13,578	Illumina Infinium Immunochip array
IGAS East Asian cohort	1,550	1,567	Illumina Infinium Immunochip array
Chinese cohort	497	4,416	Illumina HumanOmniZhongHua-8 BeadChip
Turkish cohort	1,001	1,011	Illumina CoreExome chip
Iranian cohort	479	830	Illumina CoreExome chip
Total	12,596	21,402	

*IGAS, International Genetics of Ankylosing Spondylitis Consortium.*

### Genetic Score and Exposure Assessment

To measure the magnitude of genetically proxied inhibition of HMG-CoA reductase (HMGCR), we constructed a genetic score including single nucleotide polymorphisms (SNPs) within a ± 100 kb window from HMGCR that were significantly associated with the LDL cholesterol level (*P* value < 5.0 × 10^–8^). A second inclusion criterion included the fact that all SNPs of the score were only in a weak pairwise linkage disequilibrium (*r*^2^ ≤ 0.2) in both European as well as East Asian populations. Four SNPs (rs12916, rs17648288, rs3857388 and rs10064936) that met these criteria were included in the HMGCR score as the primary exposure in this study to proxy for the inhibition of HMG-CoA reductase. The exposure alleles for each SNP were as those in association with a lower LDL cholesterol level, and their per allele effect on LDL cholesterol decrement was obtained from the GWAS meta-analysis as reported by the Global Lipids Genetics Consortium (Willer et al., 2013). For each individual, exposure of the HMGCR score was equivalent to the value by summing the number of exposure alleles inherited at each SNP multiplied by its corresponding conditional effect on the LDL cholesterol level. Using the similar criteria, we constructed the PCSK9 and NPC1L1 genetic scores composed of weakly correlated SNPs (*r*^2^ ≤ 0.2) that were associated with LDL cholesterol levels (*P* value < 5.0 × 10^–8^) and located ± 100 kb from PCSK9 and NPC1L1, respectively. In addition, we constructed the LDL polygenetic score by combining independently inherited SNPs (*r*^2^ < 0.001) from different gene regions in association with LDL cholesterol levels (*P* value < 5.0 × 10^–8^), as a natural indicator to proxy for lifelong stable LDL cholesterol levels. Differences in the genetically proxied inhibition of PCSK9 and NPC1L1 as evaluated by PCSK9 and NPC1L1 scores and genetically determined serum LDL cholesterol levels by the LDL score were defined as secondary exposure in this study.

### Study Design and Outcomes

The objective of this study is to evaluate the association of genetic scores with the risk of ankylosing spondylitis. Because inheritance of an exposure allele at each genetic variant is approximately random at birth and can exert a long-term effect throughout the lifetime of an individual, exposure differences in a combined genetic score by virtue of low linkage disequilibrium among each variant would also be naturally randomized (Palmer et al., 2012). Therefore, according to a paradigm termed as Mendelian randomization (Supplementary Figure 1), the genetic score variable might have an independent effect on events that occurred incident to the genetic exposure from the perspective of the time of birth, which is analogous to the intention-to-treat effect in a randomized controlled trial (Burgess et al., 2018). In this study, the primary outcome was the odds ratio (OR) for ankylosing spondylitis, estimated by an association with the HMGCR score. Secondary outcomes were the OR for ankylosing spondylitis associated with the PCSK9, NPC1L1 and LDL genetic scores. Exploratory outcomes included the OR for coronary artery disease and type 2 diabetes as the positive control outcomes, for which a causal effect had already been established in previous studies explained by statin therapy or genetically proxied inhibition of HMG-CoA reductase (Cholesterol Treatment Trialists’ Collaboration et al., 2010; Ference et al., 2015; Swerdlow et al., 2015). We obtained effect estimates for individual SNPs in each genetic score by performing a meta-analysis of summary data across available GWAS datasets. Data for indirectly genotyped SNPs were obtained from imputation or from a suitable proxy SNP in strong linkage disequilibrium (*r*^2^ ≥ 0.9) with reference to 1000 Genomes Phase 3 panel in the same population (1000 Genomes Project Consortium et al., 2015). The overall estimates for genetic scores were produced by combining the effect of individual SNPs using summary-level data in an inverse-variance weighted model that had taken into account weak correlation caused by the low pairwise linkage disequilibrium among variants included in genetic scores, which can be largely equivalent to effect estimates using individual-level data (Burgess et al., 2016).

### Statistical Analyses

*F* statistics were calculated to examine the strength of association between the genetic variants and the exposure factor (Palmer et al., 2012). An F statistic greater than 10 indicates a valid genetic instrument having sufficient relevance with the exposure factor (Palmer et al., 2012). The statistical power was calculated as previously described (Burgess, 2014).

With the summary-level data, we obtained the proportional effects of variants on the outcome relative to their effects on LDL cholesterol reductions using the Wald type ratio method (Burgess et al., 2013). Given the fact that a few variants were selected from the same gene region in the HMGCR, PCSK9, and NPC1L1 scores, a fixed-effects inverse-variance weighted model was utilized to combine individual SNP effects into these weighted scores, with adjustment for the weak correlations between variants (Burgess et al., 2016). In terms of potential heterogeneity from multiple LDL-cholesterol-lowering variants, a multiplicative random-effects inverse-variance weighted model was utilized for the LDL polygenetic score (Burgess et al., 2013). With the individual-level data, we evaluated the dose-response relationship between the HMGCR score and the odds ratio for ankylosing spondylitis using restricted cubic splines by remodeling the logistic regression equation. All estimates were described as odds ratios (ORs) or mean effect sizes (log OR) with 95% confidence intervals (CIs) standardized for per mmol/L decrement in LDL cholesterol levels.

For inverse-variance-weighted estimates, heterogeneity was quantified using Cochran’s Q and *I*^2^ statistics. Several sensitivity analyses were performed to examine the presence of pleiotropy meaning that genetic variants may influence the outcome *via* other pathways and not only dependent on the drug target or exposure factor of interest. For the genetic score of drug targets, we examined whether variants included in the score were associated with potential factors that had been known to affect the risk or disease activity of ankylosing spondylitis. The major risk factors previously reported for ankylosing spondylitis include male gender (Lin and Gong, 2017), HLA-B27 antigen (Lin and Gong, 2017), serum 25-hydroxyvitamin D levels (Cai et al., 2015), smoking (Videm et al., 2014), drinking (Min et al., 2019), and physical activity (O’Dwyer et al., 2017). A detectable significant association (*P* value < 0.05) between the genetic score and any of these risk factors indicated the presence of an underlying horizontal pleiotropy. Therefore, a multivariable analysis was performed to determine the independent effect of the genetic score on ankylosing spondylitis with adjustment for the effects of those significantly associated traits as covariates. For the LDL score composed of polygenetic variants, the MR Egger regression was performed to detect the unbalanced pleiotropy among multiple variants (Bowden et al., 2015). The presence of directional pleiotropy would be inferred, provided that an intercept in the regression equation was statistically different from null (zero) (*P* value < 0.05). In addition, sensitivity analyses including maximum likelihood, weighted median and MR-PRESSO outlier-corrected models were also conducted to test the robustness of the results, under different statistical assumptions addressing the bias and pleiotropy (Burgess et al., 2013; Bowden et al., 2016; Verbanck et al., 2018).

To provide complementary evidence supportive of the linkage of two traits through shared causal variants, we performed the Bayesian statistical framework colocalization analyses to prioritize genetic variants located ± 100 kb from HMGCR that may influence both the molecular trait of HMG-CoA reductase and the risk of ankylosing spondylitis (Supplementary Figure 1) (Giambartolomei et al., 2018). The per SNP posterior probability (PPA) as a shared variant between the two traits was calculated to assess whether the association between two traits were driven by different causal variants correlated due to linkage disequilibrium. In addition, given the fact that the GWAS meta-analysis by the Global Lipids Genetics Consortium was generated primarily in a European population, we performed subgroup analyses for association with ankylosing spondylitis only involving participants of European ancestry. This analysis was used to examine potential bias caused by population stratification, in which unevenly distributed genotypes across geographical regions may cause spurious association with both the ancestry and the outcome. Given the fact that the functional evidence relating genetic variants to the expression of HMGCR is currently lacking with the exception of rs12916 (Swerdlow et al., 2015; Würtz et al., 2016), we performed a sensitivity analysis only including rs12916 and removing the other variants from the HMGCR score to test its association with the risk of ankylosing spondylitis.

As the primary outcome for the HMGCR score, a significance threshold was set at *P* value < 0.05. For secondary outcomes, Bonferroni correction was applied for multiple testing, whereby a *P* value < 0.017 [0.05/3 tests (PCSK9, NPC1L1 and LDL scores)] was considered to indicate statistical significance. There was no plan on adjustment for multiple comparisons for other outcomes or in sensitivity models, and therefore these results should be interpreted as exploratory. All statistical tests were conducted as two-sided with the use of Stata version 15.0 (StataCorp, College Station, TX, United States) and R version 3.6.3 (R Foundation, St. Louis, MO, United States). For extended methods, detailed descriptions are provided in the Supplementary Appendix.

## Results

### Sample Size and Genetic Scores

Of 33,998 participants in the primary cohort, 12,596 individuals had been diagnosed with ankylosing spondylitis and 21,402 were unaffected participants (Table 1). Up to 22,233 cases with coronary artery disease and 64,762 controls as well as 62,892 type II diabetes cases and 596,424 controls from two meta-analyses of GWAS were included in the exploratory analysis for the outcome on coronary artery disease and type II diabetes, respectively. The GWAS datasets and sample size used for the covariate and multivariate analyses are summarized in Supplementary Table 1.

Four SNPs were included in the HMGCR score, eight SNPs in the PCSK9 score, two SNPs in the NPC1L1 score, and 67 SNPs in the LDL polygenetic score (Table 2 and Supplementary Table 2). Each of the chosen SNPs as well as the combined scores were strongly associated with the trait of serum LDL cholesterol levels with all F statistics greater than 10.0, respectively (Supplementary Table 3). This indicated that these scores could be used as an appropriate instrument as the genetically proxied drug target inhibition or lifelong LDL cholesterol levels.

**TABLE 2:** Effects of individual LDL cholesterol lowering variants included in HMGCR, PCSK9 and NPC1L1 genetic scores.

SNP	Location^‡^	Effect allele	Effect size (95% CI), mmol/L *	*P* value
**HMGCR genetic score**				
rs12916	chr5:74656539	T	−0.073 (−0.081 to −0.066)	7.79 × 10^–78^
rs17648288	chr5:74696638	C	−0.057 (−0.069 to −0.045)	4.60 × 10^–20^
rs3857388	chr5:74620377	T	−0.042 (−0.054 to −0.031)	2.20 × 10^–11^
rs10064936	chr5:74536723	C	−0.035 (−0.047 to −0.023)	1.78 × 10^–9^
**PCSK9 genetic score**				
rs11206510	chr1:55496039	C	−0.083 (−0.093 to −0.073)	2.38 × 10^–53^
rs2479409	chr1:55504650	A	−0.064 (−0.072 to −0.056)	2.52 × 10^–50^
rs585131	chr1:55524116	C	−0.064 (−0.074 to −0.054)	2.70 × 10^–35^
rs11206514	chr1:55516004	C	−0.051 (−0.059 to −0.043)	9.95 × 10^–33^
rs2495477	chr1:55518467	C	−0.064 (−0.075 to −0.053)	7.29 × 10^–30^
rs2479394	chr1:55486064	A	−0.039 (−0.047 to −0.031)	1.58 × 10^–19^
rs10493176	chr1:55538552	G	−0.078 (−0.098 to −0.058)	2.54 × 10^–14^
rs602705	chr1:55525726	T	−0.051 (−0.064 to −0.037)	5.06 × 10^–13^
**NPC1L1 genetic score**				
rs2073547	chr7:44582331	A	−0.049 (−0.058 to −0.039)	1.92 × 10^–21^
rs217386	chr7:44600695	A	−0.036 (−0.044 to −0.029)	1.20 × 10^–19^

*SNP, single nucleotide polymorphism.*

*^‡^Locations on hg19.*

*Effect sizes are presented as mean difference in LDL cholesterol (mmol/L) per effect allele, with 95% CIs. To convert mmol/L to mg/dL, multiply by 38.7.

For the primary outcome, assuming that the per mmol/L decrement in LDL cholesterol by the HMGCR score resulted in a twofold decrease in the odds of ankylosing spondylitis, the current sample size of the primary cohort would have a 97.4% power to obtain an estimate with a two-sided significance level of 0.05 (Supplementary Figure 2).

### Primary Outcome

**FIGURE 1:**
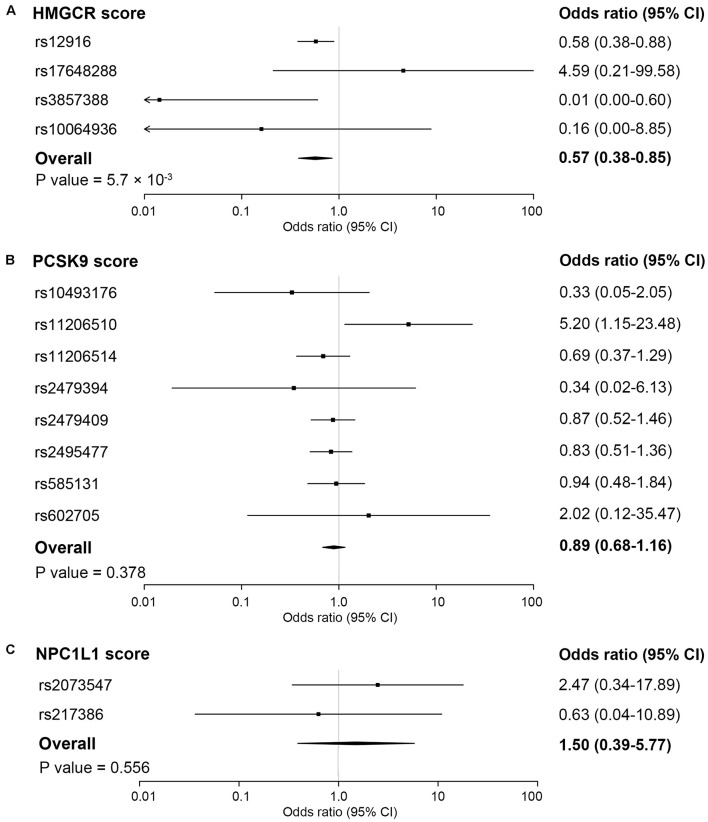
Association of genetic scores with risk of ankylosing spondylitis. The pooling effect of genetic scores of HMG-coenzyme A reductase (HMGCR) **(A)**, proprotein convertase subtilisin/kexin type 9 (PCSK9) **(B)** and NPC1 like intracellular cholesterol transporter 1 (NPC1L1) **(C)** by combining all the estimates of individual variants using inverse-variance weighted fixed-effects models, adjusted for weak linkage disequilibrium between genetic variants. Associations are scaled to a 1 mmol/L (38.7 mg/dL) reduction in LDL cholesterol. Boxes represent point estimates of odds ratio. Lines represent 95% CIs.

**FIGURE 2:**
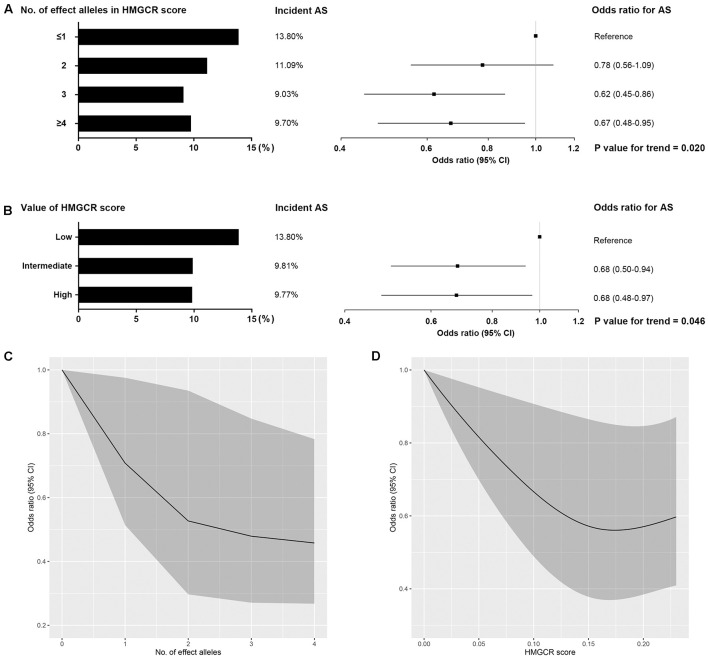
Dose-response relationship between HMGCR genetic score and risk of ankylosing spondylitis. Effect of the number of inherited LDL cholesterol-lowering alleles (effect alleles) included in HMGCR score **(A,C)** and the value of the HMGCR score **(B,D)** on the risk of ankylosing spondylitis (AS). For each person, the value of the HMGCR genetic score was calculated by adding the number of effect alleles that the person had inherited multiplied by the effect of that variant on LDL cholesterol levels. HMGCR scores were stratified by value as low (lowest quintile), intermediate (quintiles 2–4) and high (highest quintile). Bars represent the incident rate of ankylosing spondylitis in each stratification. Boxes represent point estimates of odds ratio. Lines represent 95% CIs. Odds ratios for ankylosing spondylitis per change in the number of effect alleles **(C)** and in the value of HMGCR score **(D)** were modeled with restricted cubic splines. Shaded areas show 95% CIs. These analyses were performed in the Chinese cohort involving 497 ankylosing spondylitis and 4,416 healthy controls with individual-level data.

In the inverse-variance-weighted model, genetically proxied inhibition of HMG-CoA reductase equivalent to a 1 mmol/L decrement in LDL cholesterol levels by the HMGCR score was associated with a decreased risk of ankylosing spondylitis (OR, 0.57; 95% CI, 0.38–0.85; *P* value = 5.7×10^–3^) (Figure 1). No statistically significant heterogeneity (*P* value for Q statistic = 0.121) was detected in the estimate (Supplementary Table 4). In a dose-response analysis using the individual-level data, an overall trend for a progressive reduction in risk of ankylosing spondylitis was observed when given a stepwise increase in the number of inherited exposure alleles (*P* value for trend = 0.020) or in the value of the HMGCR score (*P* value for trend = 0.046) (Figure 2). This finding suggested a dose-dependent association between genetically proxied inhibition of HMG-CoA reductase and the risk of ankylosing spondylitis.

### Secondary Outcomes

**FIGURE 3:**
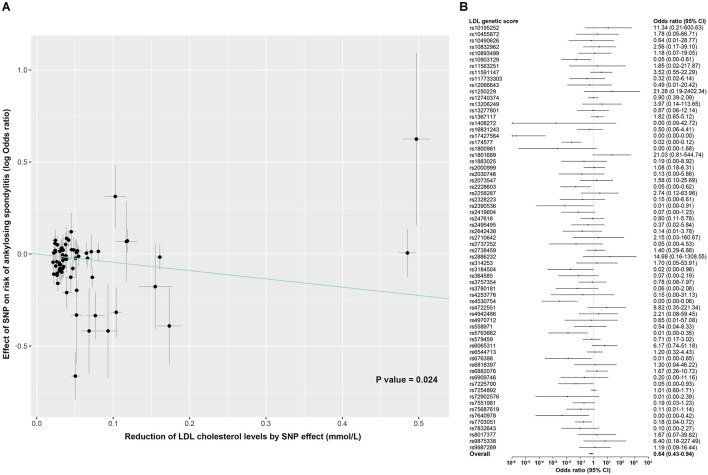
Association of LDL genetic score with risk of ankylosing spondylitis. **(A)** Scatterplot showing the effect of each SNP included in the LDL genetic score on the risk of ankylosing spondylitis against each SNP effect on the reduction of LDL cholesterol levels. Combining all SNPs as the LDL polygenetic score, a linear effect of a given reduction in LDL cholesterol levels on the risk of ankylosing spondylitis was estimated using inverse-variance weighted random-effects models. **(B)** Forest plot shows association of genetic variants included in the LDL polygenetic score with the risk of ankylosing spondylitis, individually and as weighted genetic score. Associations are scaled to a 1 mmol/L (38.7 mg/dL) reduction in LDL cholesterol. Boxes represent point estimates of odds ratio. Error bars represent 95% CIs.

No significant associations with ankylosing spondylitis were observed for the PCSK9 score (OR, 0.89; 95% CI, 0.68–1.16; *P* value = 0.378) and the NPC1L1 score (OR, 1.50; 95% CI, 0.39–5.77; *P* value = 0.556) (Figure 1). For the LDL score, the genetically determined lower LDL cholesterol level was associated with a reduced risk of ankylosing spondylitis, whereby the odds ratio was 0.64 (95% CI, 0.43–0.94; *P* value = 0.024) standardized for per mmol/L decrease in LDL cholesterol levels (Figure 3). However, when accounting for multiple testing across secondary outcomes by the Bonferroni correction, the statistical significance of the association was lost [*P* value > 0.017 (0.05/3 tests)]. In addition, a detectable heterogeneity was presented across estimates for multiple SNPs included in the LDL score (*P* value for Q statistic = 3.7 × 10^–8^) (Supplementary Table 4), indicative of the underlying presence of directional pleiotropy (Supplementary Figure 3). The MR Egger intercept test also demonstrated an unbalanced pleiotropy in the effect estimate for the LDL polygenetic score (MR Egger intercept = −0.035; *P* value = 0.036) (Supplementary Table 4).

### Exploratory Outcomes and Sensitivity Analyses

The male gender related to Chromosome Y and the HLA-B27 antigen encoded on Chromosome 6 have been reported as strong risk factors for ankylosing spondylitis, but are independent of HMGCR genetic variants located on Chromosome 5. Analyses of a subgroup of HLA-B27-antigen-tested participants also showed no association of HMGCR score with HLA-B27 antigen positivity or male gender (Supplementary Table 5). When examining the association with other known risk factors for ankylosing spondylitis, per change in HMGCR score was found to be significantly associated with a decrease in serum 25-hydroxyvitamin D levels and an increase incidence in smoking, indicating that genetic variants in the HMGCR score were likely to affect the outcome of ankylosing spondylitis *via* other pathways by influencing these two traits (Table 3). After adjusting the observed association for 25-hydroxyvitamin D level and smoking in the multivariate analyses, the odds ratio for ankylosing spondylitis per change in HMGCR score scaled to a 1 mmol/L decrement in LDL cholesterol levels was 0.12 (95% CI, 0.04–0.39) (Table 3). Similar results between the primary analysis and this sensitivity analysis demonstrated the absence of substantial bias caused by the putative horizontal pleiotropy.

**TABLE 3:** Effect of HMGCR genetic score on known potential risk factors for ankylosing spondylitis and multivariable analyses with adjustment for significantly associated risk factors.

Factor	Scale of effect size	Estimate effect of HMGCR score on risk factors		Multivariable analyses of adjusted effect on risk of ankylosing spondylitis	
		Effect size (95% CI) *	*P* value^†^	Effect size (95% CI) ^‡^	*P* value
HMGCR score	log (OR)	-	-	−2.113 (−3.290 to −0.937)^§^	4.32 × 10^–4^
Serum 25-hydroxyvitamin D levels	SD	−0.084 (−0.139 to −0.028)	0.003	30.681 (15.960 to 45.403)	4.73 × 10^–14^
Smoking initiation (ever regular)	log (OR)	0.029 (0.002 to 0.056)	0.034	36.408 (32.074 to 40.742)	1.54 × 10^–16^
HLA-B27 antigen positivity	log (OR)	−0.030 (−0.528 to 0.470)	0.919	-	-
Male gender	log (OR)	0.068 (−0.545 to 0.663)	0.837	-	-
Drinking per week	SD	0.005 (−0.049 to 0.059)	0.849	-	-
Overall physical activity time	SD	−0.017 (−0.137 to 0.103)	0.777	-	-

*log (OR), log transformed odds ratio; SD, standard deviation.*

*Effect sizes are presented per unit change in HMGCR score equivalent to a 1 mmol/L (38.7 mg/dL) reduction in LDL cholesterol.

*^†^A *P* value < 0.05 indicated the presence of an association of the HMGCR genetic score with the corresponding risk factor for ankylosing spondylitis, for which was then adjusted in multivariable analyses to examine the independent association between the HMGCR genetic score and the risk of ankylosing spondylitis.*

*^‡^Effect sizes are presented per unit change in HMGCR score or in the corresponding risk factors genetically modified by a combined effect of rs12916, rs17648288, rs3857388 and rs10064936.*

*^§^The odds ratio for ankylosing spondylitis transformed from the effect size per change in HMGCR score equivalent to a 1 mmol/L reduction in LDL cholesterol was 0.12 (95% CI, 0.04–0.39), after adjustment for the associations of serum 25-hydroxyvitamin D levels and smoking with the risk of ankylosing spondylitis.*

For the LDL polygenetic score, several different statistical models were used to adjust for potential bias and pleiotropy (Supplementary Table 6). Directionally consistent estimates on the risk of ankylosing spondylitis were obtained by maximum likelihood (OR, 0.63; 95% CI, 0.49–0.83), MR Egger regression bootstrap (OR, 0.78; 95% CI, 0.47–1.29), weighted median (OR, 0.98; 95% CI, 0.64–1.49) and MR-PRESSO outlier-corrected (OR, 0.75; 95% CI, 0.54–1.03) models, but with wider confidence intervals.

Evidence of colocalization across variants nearby HMGCR proxying HMG-CoA reductase inhibition and ankylosing spondylitis with a posterior probability (PPA) > 0.01 was detected for three SNPs, rs7703051, rs11749783 and rs3846663, of which all are in strong linkage disequilibrium with each other (*r*^2^ = 1.0) (Supplementary Figure 4). The top-colocalized SNP rs7703051 (PPA = 0.515) is also in high linkage disequilibrium with a variant (rs12916) included in the HMGCR score (*r*^2^ = 0.98). The effect estimate for rs7703051 was consistent with the primary analysis in an odds ratio of 0.62 (95% CI, 0.40–0.98) for ankylosing spondylitis proportional to per mmol/L decrement in LDL cholesterol levels. These findings implied that there might be a biological mechanism linking HMG-CoA reductase inhibition and ankylosing spondylitis *via* a shared causal locus within HMGCR. The observed association between the two traits was thus less likely to be driven by different gene regions.

Furthermore, subgroup analyses only including the European population provided a similar estimate for the HMGCR score (OR, 0.56; 95% CI, 0.36–0.85) as well as other genetic scores, as compared with the results from the full primary cohort (Supplementary Table 7). Therefore, these data suggested that when combining different populations to maximize the sample size and statistical power, the primary analysis did not suffer from substantial bias influenced by population stratification.

There was functional evidence relating rs12916 to the mRNA expression of HMGCR, supporting its validity as genetic instruments in our analysis (Swerdlow et al., 2015; Würtz et al., 2016). However, despite the strong association of the selected SNPs with the LDL cholesterol level (*P* value<5.0×10^–8^), the biological function of the other genetic variants included in HMGCR score is currently unclear. A sensitivity analysis leaving out the other SNPs and only including rs12916 in the HMGCR score showed a consistent association with the risk of ankylosing spondylitis (OR, 0.58; 95% CI, 0.38–0.88) as compared with the primary analysis.

For exploratory outcomes, inverse-variance-weighted analyses showed a decreased risk for coronary artery disease as well as an increased risk for type 2 diabetes especially associated with genetic variants related to genetically proxied inhibition of HMG-CoA reductase (Supplementary Table 8). These results were in line with expectation based on the protective role of statins on coronary artery disease and a higher incidence of type II diabetes in statin-treated patients (Cholesterol Treatment Trialists’ Collaboration et al., 2010; Ference et al., 2015; Swerdlow et al., 2015), which suggested that genetic variants included in the HMGCR score can reliably assess the effect of HMG-CoA reductase inhibition.

## Discussion

This study showed that genetic variants proxying for HMG-CoA reductase inhibition were protective for ankylosing spondylitis. These variants appeared to mimic the pharmacological effect of statins, as evidenced by having a similar effect to long-term statin therapy in modifying the risk of coronary artery disease and type 2 diabetes (Cholesterol Treatment Trialists’ Collaboration et al., 2010; Ference et al., 2015; Swerdlow et al., 2015). These findings highlighted the potential of statins in the treatment and prevention of ankylosing spondylitis. Our findings did not show sufficient evidence on the association of genetically proxied inhibition of PCSK9 or NPC1L1 as well as genetically determined LDL cholesterol levels with the risk of ankylosing spondylitis.

Statins have similarly shown benefits for patients with ankylosing spondylitis in previous reports (van Denderen et al., 2006; Garg et al., 2015; Rollefstad et al., 2015; van der Valk et al., 2016; Oza et al., 2017). In a population-based cohort, initiation of statins was associated with a remarkable reduction in the mortality of ankylosing spondylitis (Oza et al., 2017). Patients with ankylosing spondylitis often have an excessive cardiovascular risk, probably due to increased arterial wall inflammation as well as the frequent use of immunomodulatory drugs that could increase cholesterol levels (Haroon et al., 2015; Semb and Rollefstad, 2016). Sustained improvement in arterial wall inflammation, endothelial function and atherosclerosis was observed in statin-treated patients with ankylosing spondylitis or other inflammatory joint diseases (Ikdahl et al., 2015; Rollefstad et al., 2015; van der Valk et al., 2016). Moreover, statins not only play an anti-inflammatory role in the cardiovascular system, but can also relive the symptoms of spondylitis accompanied by attenuation of systemic inflammatory biomarkers, as shown in an open pilot trial of 12-week treatment with Rosuvastatin (van Denderen et al., 2006). In addition, a randomized trial involving 32 patients showed that 24-week treatment with Rosuvastatin led to improvement in endothelial dysfunction and clinical disease activity of ankylosing spondylitis, as compared with the placebo group (Garg et al., 2015). However, there was a discrepancy between observational studies and randomized clinical trials in rheumatic diseases (Jiang et al., 2018). Observational analyses showed a significant decrease in mortality with statin use in rheumatic diseases, however, randomized trials demonstrated limited effectiveness of statins in these patients (Jiang et al., 2018). This might be due to the fact that observational analyses can be affected by immortal time bias, and therefore a well-designed randomized controlled trial is currently needed (Jiang et al., 2018). One should be cautious that data from a confirmatory phase 3 trial of statin therapy for ankylosing spondylitis are currently lacking. Our study did not provide direct evidence indicative of risk reduction in ankylosing spondylitis from statin use, and results of this study along with previous findings only suggested that the HMG-CoA reductase mediated pathway is implicated in the pathogenesis of ankylosing spondylitis and that HMG-CoA reductase could be a possible drug target for the prevention and treatment of ankylosing spondylitis.

Furthermore, on the basis of human genetic evidence in ankylosing spondylitis, a common inflammatory disease, our findings also support the hypothesis that inhibition of HMG-CoA reductase may produce an independent anti-inflammatory effect beyond its lipid-lowering activity. The lack of a significant association with PCSK9 score, NPC1L1 score and LDL polygenetic score may imply that simply lowering serum cholesterol levels would be unlikely to contribute to the prevention of ankylosing spondylitis. Experimental *in vitro* studies or in animal models have shown that the anti-inflammatory feature of statins could modify not only atherogenesis but also tissues and immune cells involved in numerous inflammatory processes, such as suppressing the pro-inflammatory mediators including TNF-α, IL-1β, and IL-6 and inhibiting the overreacted Th1 and Th17 response (Chalubinski and Broncel, 2010). There were accumulated studies showing that statins exhibited pleiotropic properties beyond lowering the LDL cholesterol levels (Nussenblatt et al., 1985; Antonopoulos et al., 2012; Jiang et al., 2018). Inhibition of HMG-CoA reductase by statins was found to decrease the cholesterol synthesis and isoprenoid production, which led to the prenylation of small G-proteins such as Rho and, in turn, reduced the activation of pro-inflammatory nuclear factor-κB (NF-κB) signaling pathway (Antonopoulos et al., 2012). The observation on statin-mediated inhibition of Rho might be a possible explanation for their anti-inflammatory effects, and our study provided genetic evidence in populations consistently suggesting the anti-inflammatory effects of HMG-CoA reductase inhibition in ankylosing spondylitis. Nevertheless, further studies are required to clarify the precise mechanisms involved.

This study has several strengths. First, findings of this study may have some clinical relevance. From the current study together with existing reports, it would be reasonable to expect that statin therapy might be a promising strategy to prevent the progression of ankylosing spondylitis as well as its associated complications. Second, by virtue of the natural allocation of genetic variants from the perspective of the time of birth, this study provides nearly randomized evidence to validate and prioritize the drug targets in human beings, which can minimize the possibility of residual confounding and reverse causality. In addition, with diverse analytic methods to address the potential pleiotropy and confounding as far as we can, including multivariable analyses, dose-response analyses, colocalization, subgroup analyses and introducing the positive control outcomes, this study has presented robust estimates for the primary outcome.

This study has some major limitations. First, the estimated effect in this study could not be straightly translated into the effect size in the real-world, because the genetic variants exert a lifelong profound regulation on the drug target and may lead to a more intensified effect as compared with that from a short-period pharmacological intervention. On the other hand, the inhibitory effect of genetic variants on the drug target was not directly quantified but was scaled in accordance with the decrement in LDL cholesterol levels, which was based on the speculation that assumes drug target inhibition proportional to the decrease in LDL cholesterol levels. A similar approach had been used previously (Yarmolinsky et al., 2020). Moreover, according to our selection criteria (also similar with the previous study) (Yarmolinsky et al., 2020), variants strongly associated with the LDL cholesterol levels were included in the genetic score, however, the biological function of most genetic variants is currently unclear. Therefore, our study may be limited by the validity of selected genetic instruments. Nevertheless, a similar association between genetically proxied HMGCR inhibition and the risk of ankylosing spondylitis was observed when only a functional variant rs12916 was used to proxy for the inhibition of HMGCR. Second, the covariate information regarding statin use was lacking in the dataset source we used, and we therefore could not perform a further adjustment for statin use. By virtue of the natural allocation of genetic variants from the perspective of the time of birth (Burgess et al., 2012), Mendelian randomization is similar to a naturally occurring randomized controlled trial to minimize confounding bias and is particularly useful for studies in which certain covariate information is unmeasured as is the case with our study. Third, data on the follow-up and prognosis of ankylosing spondylitis, especially disease activity, cardiovascular events and death, were not available in the GWAS datasets we used. Cautious interpretation should therefore be made from this study regarding the therapeutic effect of statins on ankylosing spondylitis, and as mentioned above, well powered trials designed with a suitable outcome are necessary to evaluate the long-term efficacy and safety of statins on the disease. Fourth, in terms of secondary outcomes, no significant associations were detected for PCSK9 and NPC1L1 scores, which may be due to insufficient statistical power. The statistical powers for PCSK9 and NPC1L1 scores were less than 80%, when assuming an odds ratio of 0.9 and 1.5 expected per change in PCSK9 and NPC1L1 scores, respectively (Supplementary Figure 2). In addition, despite the fact that the association has been tested with different assumptions in sensitivity analyses, due to the study design, we could not totally ruled out the possibly of bias especially in confounding or pleiotropy, nor could we anticipate the off-target outcomes that are not related to the mechanism of action of drugs.

In conclusion, genetically proxied inhibition of HMGCR is associated with a reduced risk of ankylosing spondylitis, which is consistent with clinical observations showing the benefits of statin use for ankylosing spondylitis. Based on data from this large-scale genetic analysis, HMG-coenzyme A reductase inhibition could provide a novel therapeutic approach to ankylosing spondylitis. This study, at least in part, provides a biological explanation for the existing observations and also indicates the direction for future large-scale trials.

## Data Availability Statement

The datasets presented in this study can be found in online repositories. The names of the repository/repositories and accession number(s) can be found in the article/Supplementary Material.

## Ethics Statement

The studies involving human participants were reviewed and approved by the Ethics Committee of the First Affiliated Hospital of Chongqing Medical University. The patients/participants provided their written informed consent to participate in this study.

## Author Contributions

PY and ZZ conceived and designed the study. ZZ, XF, GS, and WL did the literature search. ZZ, LD, SL, FL, XZ, and PY enrolled participants and collected clinical data. ZZ, XF, and XZ analyzed and interpreted the data. ZZ wrote the first draft of the manuscript. PY supervised the study. All authors provided a final review and approved the manuscript before submission.

## Conflict of Interest

The authors declare that the research was conducted in the absence of any commercial or financial relationships that could be construed as a potential conflict of interest.

## Publisher’s Note

All claims expressed in this article are solely those of the authors and do not necessarily represent those of their affiliated organizations, or those of the publisher, the editors and the reviewers. Any product that may be evaluated in this article, or claim that may be made by its manufacturer, is not guaranteed or endorsed by the publisher.
