# Supplementary material for: HMG-Coenzyme A Reductase as a Drug Target for the Prevention of Ankylosing Spondylitis
Source: Front Cell Dev Biol. 2021 Oct 6;9:731072. doi: 10.3389/fcell.2021.731072 (PMC8526849; doi:10.3389/fcell.2021.731072)
Supplement: Supplementary file 1 [file Data_Sheet_1.docx]

**Supplementary Appendix**

[Supplementary Online Text Extended analytic methods 2](#_Toc43282847)

[Supplementary Online Text Data sources and acknowledgements 6](#_Toc43282848)

[Supplementary Table S1. Summary of sample size used in the covariate and multivariate analyses for meta-analysis of association of HMGCR variants with potential covariates 11](#_Toc43282849)

[Supplementary Table S2. Effects of individual variants included in LDL genetic score on LDL cholesterol levels 12](#_Toc43282850)

[Supplementary Table S3. Strength of association between genetic scores and LDL cholesterol level phenotypes 14](#_Toc43282851)

[Supplementary Table S4. Heterogeneity statistics in association estimates and directional pleiotropy detected by the MR Egger regression 15](#_Toc43282852)

[Supplementary Table S5. Association of HMGCR genetic score with HLA-B27 positivity and gender 16](#_Toc43282853)

[Supplementary Table S6. Analyses of association between genetically determined LDL cholesterol levels and risk of ankylosing spondylitis using different estimation models 17](#_Toc43282854)

[Supplementary Table S7. Comparison of estimates for association with risk of ankylosing spondylitis between the primary cohort and the European population 18](#_Toc43282855)

[Supplementary Table S8. Association of genetic variants related to inhibition of drug target with risk of coronary artery disease and type Ⅱ diabetes 19](#_Toc43282856)

[Supplementary Figure S1. Pattern diagrams illustrating causal inferences in mendelian randomisation and colocalisation 20](#_Toc43282857)

[Supplementary Figure S2. Statistical power calculations for primary analyses 21](#_Toc43282858)

[Supplementary Figure S3. Funnel plot of genetic variants included in the LDL genetic score 22](#_Toc43282859)

[Supplementary Figure S4. Colocalisation analysis of genetic variants in HMGCR 23](#_Toc43282860)

[References 24](#_Toc43282861)

Supplementary Online Text Extended analytic methods

**Genetic score**

For genetic scores of drug targets, we obtained single nucleotide polymorphisms (SNPs) with a minor allele frequency (MAF) of at least 1% within a ±100 kb window from the gene encoding each drug target and associated with the LDL cholesterol level at P value < 5.0 × 10^−8^. To further ensure an approximate independence of included SNPs, we applied a linkage disequilibrium clumping approach to retain the most significant SNP (with the lowest P value) per linkage disequilibrium block, removing other SNPs in that block in linkage disequilibrium with the most significant SNP (r^2^ > 0.2) in both European and East Asian populations with reference to 1000 Genomes Phase 3 panel. The exposure alleles for each SNP were as those in association with a lower LDL cholesterol level, and their per allele effect on LDL cholesterol decrement was obtained from the GWAS meta-analysis as reported by the Global Lipids Genetics Consortium.[^1^](#_ENREF_1) For each individual, exposure of the HMGCR score was equivalent to the value by summing the number of exposure alleles inherited at each SNP multiplied by its corresponding conditional effect on the LDL cholesterol level.

For the polygenetic LDL score, a similar approach was applied, but we included SNPs with MAF ≥ 0.01, in association with the LDL cholesterol level at P value < 5.0 × 10^−8^, irrespective of genomic location of SNPs, and with the removal of other SNPs in each block in linkage disequilibrium with the most significant SNP (r^2^ > 0.001).

**Inverse-variance-weighed estimates for correlated variants**

The Wald type ratio method was used to provide the individual estimate for each SNP through dividing the effect size on the study outcome by the effect size of that allele of the SNP on the LDL cholesterol levels, whereby the standard error was calculated by the delta method approximation.[^2^](#_ENREF_2) An inverse-variance-weighted model was generated to evaluate the pooling effect size from each SNP included in the genetic score. This regression model is based on the meta-analysis principle to produce a weighted average of the individual genetic associations without an intercept term. Given the fact that variants in the genetic score of drug targets were in weak linkage disequilibrium with each other, we modified the inverse-variance-weighted model into a generalised linear regression with further adjustment for the partial correlation between variants included in each genetic score, as previously described. [^3^](#_ENREF_3)

**MR Egger regression**

For the LDL polygenetic score, the MR Egger regression was performed as a sensitivity analysis to detect the unbalanced pleiotropy among multiple variants. The MR Egger regression is based on the assumption allowing an overall unbalanced pleiotropy among genetic variants.[^4^](#_ENREF_4) The regression equation provides two parameters, including the slope interpreted as the regression coefficient adjusted for the overall pleiotropy and the intercept term as the horizontally pleiotropic effect. Therefore, if the intercept term is significantly different from null (zero) (P < 0.05), there will be directional horizontal pleiotropy that has not averaged to zero in the estimates across multiple genetic variants. However, the effect size obtained from the MR Egger regression is usually considered to be underpowered.

**Weighted median estimator**

The weighted median regression was performed as a complementary analysis for the LDL polygenetic score, as previously described.[^5^](#_ENREF_5) This approach assumes a weighted median of a distribution where each estimate of variants are ordered and weighted by the reciprocal of the variance of the ratio estimate. This estimator would provide an unbiased causal estimate even if up to a half of the information came from invalid genetic variants. This method would have improved finite-sample Type 1 error rates than the inverse-variance-weighted model, and would provide a higher precision than that from the MR Egger regression.

**MR-PRESSO outlier-corrected model**

Mendelian Randomisation Pleiotropy RESidual Sum and Outlier (MR-PRESSO) test is another approach to identify and address the horizontal pleiotropy.[^6^](#_ENREF_6) The MR-PRESSO outlier test can be used to detect the presence of specific horizontal pleiotropic outlier SNP by using the observed and expected distributions of the tested SNP. With the removal of the significant horizontal pleiotropic outlier SNPs, the MR-PRESSO outlier-corrected model could provide an unbiased causal estimate and would be most suitable when horizontal pleiotropy occurs in <50% of SNPs. We applied this model as a sensitivity analysis for the LDL polygenetic score.

**Maximum likelihood model**

A maximum likelihood based estimate was performed as a sensitivity analysis, as previously described.[^2^](#_ENREF_2) The likelihood based model provides an estimate for a linear relationship between the exposure and the outcome, whereby the exposure and the outcome are assumed to be jointly under a bivariate normal distribution. The maximum likelihood approach could provide less biased estimates over the inverse-variance-weighed model even if the gene-exposure and the gene-outcome associations are correlated or when using a weak instrument of which associations of genetic variants and the exposure are not as precisely measured.

Supplementary Online Text Data sources and acknowledgements

***International Genetics of Ankylosing Spondylitis Consortium*** [***^7^***](#_ENREF_7)

International Genetics of Ankylosing Spondylitis Consortium, along with ustralo-Anglo-American Spondyloarthritis Consortium (tASC), Groupe Française d'Etude Génétique des Spondylarthrites (GFeGS), Nord-Trøndelag Health Study (HUNT), Spondyloarthritis Research Consortium of Canada (SPARCC) and Wellcome Trust Case Control Consortium 2 (ETCCC2) published the results of a collaborative research on the genome-wide association study (GWAS) for ankylosing spondylitis on *Nat Genet. 2013; 45(7): 730-738*. This GWAS dataset eventually included a European cohort of 9,069 cases and 13,578 controls and an East Asian cohort of 1,550 cases and 1,567 controls. Participants enrolled in this study were from the UK 1958 Birth Cohort, the UK Blood Services Common Controls and the United States and from participating centres from France, The Netherlands, Norway, Spain, Mexico, Colombia, China, Taiwan and Korea. Summary-level datasets of the European cohort and the East Asian cohort were publicly available at https://dataverse.harvard.edu/dataset.xhtml?persistentId=doi:10.7910/DVN/NJ7XSO.

***Genome-wide association study in Turkish and Iranian populations identify rare familial Mediterranean fever gene (MEFV) polymorphisms associated with ankylosing spondylitis*** [***^8^***](#_ENREF_8)

The genome-wide association study on ankylosing spondylitis included two cohorts, the Turkish cohort composed of 1,001 Turkish ankylosing spondylitis patients and 1,011 Turkish controls, the Iranian cohort composed of 479 Iranian cases and 830 controls. Summary-level datasets of the two cohorts for the analyses in our study are available at https://dataverse.harvard.edu/dataset.xhtml?persistentId=doi:10.7910/DVN/KRDWFR.

***Genome-wide association study on ankylosing spondylitis with individual-level data***

This genome-wide association study (GWAS) on ankylosing spondylitis of the Chinese cohort was performed by our group including 497 cases with ankylosing spondylitis and 4,416 healthy controls of Han Chinese. All participants provided written informed consent. The genome-wide association study was approved by the Ethics Committee of the First Affiliated Hospital of Chongqing Medical University and adhered to the tenets of the Declaration of Helsinki. The manuscript on this GWAS is being prepared in house and the full summary statistics will be available at http://www.uvogene.com upon the publication of the GWAS.

***Global Lipids Genetics Consortium*** [***^1^***](#_ENREF_1)

Global Lipids Genetics Consortium was an international collaborative research on the genetic associations for plasma lipids. The Consortium performed a meta-analysis from 45 genome-wide association studies that were based on either a population-based study design or a case-control design, involving up to 188,578 participants. Summary results for the GWAS datasets are available at http:/www.sph.umich.edu/csg/abecasis/lipids2013/.

***CARDIoGRAM Consortium*** [***^9^***](#_ENREF_9)

Coronary ARtery DIsease Genome wide Replication and Meta-analysis (CARDIoGRAM) was an international collaboration by combining data from multiple large-scale genetic studies to identify risk loci for coronary artery disease. CARDIoGRAM Consortium released the summary dataset of meta-analysis of 22 genome-wide association studies on coronary artery disease involving up to 22,233 cases and 64,762 controls of European descent, at the website of http://www.cardiogramplusc4d.org/data-downloads/.

***Genome-wide association analyses identify 143 risk variants and putative regulatory mechanisms for type 2 diabetes*** [***^10^***](#_ENREF_10)

This genome-wide association study combined summary statistics of DIAbetes Genetics Replication And Meta-analysis (DIAGRAM), Genetic Epidemiology Research on Adult Health and Aging (GERA), and the UK Biobank by meta-analysis to investigate the genetic association with type 2 diabetes involving up to 62,892 type 2 diabetes cases and 596,424 controls of European ancestry. Summary data from the meta-analysis are available at https://cnsgenomics.com/content/data.

***Genome-wide association study in 79,366 European-ancestry individuals informs the genetic architecture of 25-hydroxyvitamin D levels*** [***^11^***](#_ENREF_11)

This study expanded the previous SUNLIGHT consortium GWAS, and conducted a large-scale, multicentre, genome-wide association analysis on serum 25-hydroxyvitamin D concentrations, involving 79,366 samples of European descent from 31 cohorts in Europe, Canada and USA. For the full descriptions of all participating cohorts, please refer to *Nat Commun. 2018; 9:260*. The GWAS summary statistics on serum circulating vitamin D concentrations are available at dbGap https://drive.google.com/drive/folders/0BzYDtCo_doHJRFRKR0ltZHZWZjQ.

***GWAS of smoking behaviour in 165,436 Japanese people reveals seven new loci and shared genetic architecture*** [***^12^***](#_ENREF_12)

This genome-wide association study investigated the genetic association with smoking behaviour in approximately 200,000 individuals who were enrolled in the BBJ Projects. The dataset involving 83,830 ever smokers and 81,626 never smokers of Japanese descent contributed to our meta-analysis of association of HMGCR variants with the trait of smoking initiation. The full GWAS summary data are available at http://jenger.riken.jp/en/.

***GWAS & Sequencing Consortium of Alcohol and Nicotine use*** [***^13^***](#_ENREF_13)

The GWAS & Sequencing Consortium of Alcohol and Nicotine use (GSCAN) was an international genetic association consortium dedicated to identification of candidate genes and their alleles that modify the phenotypes of behaviours of alcohol and nicotine uses based on millions of participants from worldwide. The dataset involving 557,337 ever smokers and 674,754 never smokers and the dataset on measures of alcohol use involving 941,280 individuals contributed to our meta-analysis of association of HMGCR variants with the trait of smoking initiation and drinking per week, respectively. GWAS summary statistics are available from the web https://genome.psych.umn.edu/index.php/GSCAN.

***GWAS identifies 14 loci for device-measured physical activity and sleep duration*** [***^14^***](#_ENREF_14)

This genome-wide association study reported genetic associations with device-measured physical activity and sleep duration in 91,105 UK Biobank participants. The dataset on measures of physical activity contributed to our meta-analysis of association of HMGCR variants with a continuous phenotype of overall activity time. GWAS summary statistics are available from https://ora.ox.ac.uk/objects/uuid:ff479f44-bf35-48b9-9e67-e690a2937b22.

Supplementary Table S1. Summary of sample size used in the covariate and multivariate analyses for meta-analysis of association of HMGCR variants with potential covariates

| **Outcome**  **(potential covariates)** | **Trait** | **No. of participants** | **No. of cases^*^** | **Population** |
| --- | --- | --- | --- | --- |
| Serum 25-hydroxyvitamin D levels | Quantitative | 79,366 | - | European ancestry |
| Smoking initiation | Binary | 1,397,547 | 641,167 | European and East Asian ancestry |
| Drinking per week | Quantitative | 941,280 | - | Worldwide (Majority of European ancestry) |
| Overall physical activity time | Quantitative | 91,105 | - | European ancestry |

^*^ Cases were those individuals with the corresponding outcome.

Supplementary Table S2. Effects of individual variants included in LDL genetic score on LDL cholesterol levels

| **SNP** | **Effect allele** | **Effect size (mmol/L) ^*^** | **Standard error of effect size** | **Sample size ^†^** | **P value** |
| --- | --- | --- | --- | --- | --- |
| rs10195252 | C | -0.0238 | 0.0039 | 157208 | 3.81 × 10^-8^ |
| rs10455872 | A | -0.1174 | 0.0143 | 84517 | 1.94 × 10^-15^ |
| rs10490626 | A | -0.0508 | 0.0069 | 173044 | 1.70 × 10^-12^ |
| rs10832962 | C | -0.0320 | 0.0040 | 172920 | 6.62 × 10^-14^ |
| rs10893499 | G | -0.0521 | 0.0053 | 172980 | 3.86 × 10^-21^ |
| rs10903129 | A | -0.0328 | 0.0037 | 169920 | 3.03 × 10^-17^ |
| rs11563251 | C | -0.0345 | 0.0062 | 172855 | 4.50 × 10-^8^ |
| rs11591147 | T | -0.4970 | 0.0180 | 77417 | 8.58 × 10^-143^ |
| rs117733303 | A | -0.1551 | 0.0220 | 77461 | 8.48 × 10^-11^ |
| rs12066643 | T | -0.0389 | 0.0064 | 171295 | 1.06 × 10-^8^ |
| rs1250229 | T | -0.0243 | 0.0042 | 173032 | 3.13 × 10-^8^ |
| rs12740374 | T | -0.1610 | 0.0044 | 172820 | 2.41 × 10^-272^ |
| rs13206249 | A | -0.0378 | 0.0062 | 87149 | 4.53 × 10^-8^ |
| rs13277801 | T | -0.0338 | 0.0038 | 173010 | 3.99 × 10^-17^ |
| rs1367117 | G | -0.1186 | 0.0040 | 173007 | 9.48 × 10^-183^ |
| rs1408272 | G | -0.0520 | 0.0083 | 167888 | 3.68 × 10^-9^ |
| rs16831243 | C | -0.0378 | 0.0055 | 162945 | 9.06 × 10^-12^ |
| rs17427564 | T | -0.0505 | 0.0058 | 153470 | 1.95 × 10^-16^ |
| rs174577 | A | -0.0523 | 0.0038 | 172952 | 1.04 × 10^-40^ |
| rs1800961 | T | -0.0685 | 0.0106 | 142698 | 6.03 × 10^-10^ |
| rs1801689 | A | -0.1028 | 0.0139 | 111143 | 9.81 × 10^-12^ |
| rs1883025 | T | -0.0296 | 0.0044 | 172330 | 6.14 × 10^-11^ |
| rs2000999 | G | -0.0650 | 0.0046 | 171510 | 4.22 × 10^-41^ |
| rs2030746 | C | -0.0214 | 0.0038 | 173024 | 8.61 × 10^-9^ |
| rs2073547 | A | -0.0485 | 0.0049 | 169889 | 1.92 × 10^-21^ |
| rs2228603 | T | -0.1040 | 0.0072 | 158643 | 4.43 × 10^-44^ |
| rs2258287 | C | -0.0328 | 0.0039 | 172889 | 6.66 × 10^-17^ |
| rs2328223 | A | -0.0299 | 0.0050 | 170762 | 5.63 × 10^-9^ |
| rs2390536 | G | -0.0223 | 0.0038 | 172981 | 2.04 × 10^-8^ |
| rs2419604 | G | -0.0302 | 0.0040 | 172807 | 7.49 × 10^-14^ |
| rs247616 | T | -0.0547 | 0.0041 | 171458 | 2.57 × 10^-37^ |
| rs2495495 | C | -0.0342 | 0.0059 | 162403 | 3.52 × 10^-8^ |
| rs2642438 | A | -0.0352 | 0.0042 | 165470 | 7.32 × 10^-16^ |
| rs2710642 | G | -0.0239 | 0.0038 | 172994 | 6.09 × 10^-9^ |
| rs2737252 | A | -0.0314 | 0.0041 | 172950 | 7.04 × 10^-14^ |
| rs2738459 | C | -0.0532 | 0.0058 | 88433 | 2.26 × 10^-19^ |
| rs2886232 | C | -0.0451 | 0.0064 | 162498 | 3.88 × 10^-11^ |
| rs314253 | C | -0.0242 | 0.0038 | 169706 | 3.44 × 10^-10^ |
| rs3184504 | T | -0.0268 | 0.0038 | 164996 | 4.20 × 10^-12^ |
| rs364585 | A | -0.0249 | 0.0038 | 171526 | 4.28 × 10^-10^ |
| rs3757354 | T | -0.0382 | 0.0044 | 172987 | 2.09 × 10^-17^ |
| rs3780181 | G | -0.0445 | 0.0074 | 171976 | 1.76 × 10^-9^ |
| rs4253776 | A | -0.0311 | 0.0059 | 171071 | 3.35 × 10^-8^ |
| rs4530754 | G | -0.0275 | 0.0036 | 173003 | 3.58 × 10^-12^ |
| rs4722551 | T | -0.0391 | 0.0049 | 172946 | 3.95 × 10^-14^ |
| rs4942486 | C | -0.0243 | 0.0037 | 171930 | 2.26 × 10^-11^ |
| rs4970712 | A | -0.0339 | 0.0044 | 173036 | 2.46 × 10^-13^ |
| rs558971 | A | -0.0382 | 0.0037 | 172992 | 5.56 × 10^-24^ |
| rs5763662 | C | -0.0767 | 0.0121 | 162777 | 1.19 × 10^-8^ |
| rs579459 | T | -0.0665 | 0.0045 | 172706 | 2.42 × 10^-44^ |
| rs6065311 | T | -0.0417 | 0.0036 | 171333 | 1.66 × 10^-30^ |
| rs6544713 | C | -0.0806 | 0.0041 | 172940 | 4.84 × 10^-83^ |
| rs676388 | T | -0.0265 | 0.0039 | 166830 | 1.31 × 10^-11^ |
| rs6818397 | G | -0.0224 | 0.0040 | 172685 | 1.68 × 10^-8^ |
| rs6882076 | T | -0.0456 | 0.0038 | 173006 | 3.31 × 10^-31^ |
| rs6909746 | T | -0.0263 | 0.0037 | 170097 | 7.86 × 10^-11^ |
| rs7225700 | T | -0.0297 | 0.0038 | 171505 | 3.56 × 10^-13^ |
| rs7254892 | A | -0.4853 | 0.0119 | 139198 | 0.00 × 10^0^ |
| rs72902576 | G | -0.0933 | 0.0133 | 82068 | 9.58 × 10^-12^ |
| rs7551981 | G | -0.0472 | 0.0038 | 173021 | 1.36 × 10^-33^ |
| rs75687619 | G | -0.1735 | 0.0161 | 82004 | 8.05 × 10^-24^ |
| rs7640978 | T | -0.0392 | 0.0069 | 172228 | 9.84 × 10^-9^ |
| rs7703051 | C | -0.0727 | 0.0037 | 173015 | 1.40 × 10^-77^ |
| rs7832643 | G | -0.0339 | 0.0038 | 164854 | 2.67 × 10^-17^ |
| rs8017377 | G | -0.0303 | 0.0038 | 172866 | 2.52 × 10^-15^ |
| rs9875338 | A | -0.0270 | 0.0037 | 172895 | 2.21 × 10^-11^ |
| rs9987289 | A | -0.0714 | 0.0066 | 160102 | 8.53 × 10^-24^ |

**^*^** Effect sizes are presented as mean difference in LDL cholesterol (mmol/L) per effect allele, with 95% CIs. To convert mmol/L to mg/dL, multiply by 38.7.

**^†^** Sample sizes used in the GWAS meta-analysis of LDL cholesterol levels by the Global Lipids Genetics Consortium.

Supplementary Table S3. Strength of association between genetic scores and LDL cholesterol level phenotypes

| **Gene** | **SNP** | **Sample size^†^** | **F-statistic ^‡^** |
| --- | --- | --- | --- |
| HMGCR | rs12916 | 168357 | 444.9 |
| HMGCR | rs17648288 | 172878 | 102.2 |
| HMGCR | rs3857388 | 172939 | 68.5 |
| HMGCR | rs10064936 | 89888 | 43.2 |
| **HMGCR genetic score** | (4 SNPs combined) | - | 155.6 |
|  | | | |
| PCSK9 | rs11206510 | 172812 | 312.2 |
| PCSK9 | rs2479409 | 172970 | 317.0 |
| PCSK9 | rs585131 | 167769 | 205.3 |
| PCSK9 | rs11206514 | 172996 | 211.7 |
| PCSK9 | rs2495477 | 80151 | 157.9 |
| PCSK9 | rs2479394 | 172953 | 105.1 |
| PCSK9 | rs10493176 | 86056 | 105.4 |
| PCSK9 | rs602705 | 89888 | 69.1 |
| **PCSK9 genetic score** | (8 SNPs combined) | - | 187.5 |
|  | | | |
| NPC1L1 | rs2073547 | 169889 | 125.0 |
| NPC1L1 | rs217386 | 173021 | 110.2 |
| **NPC1L1 genetic score** | (2 SNPs combined) | - | 117.8 |
|  | | | |
| **LDL polygenetic score** | (67 SNPs combined) | - | 197.0 |

**^†^** Sample sizes used in the GWAS meta-analysis of LDL cholesterol levels by the Global Lipids Genetics Consortium.

**^‡^** The F statistic is a measure of instrument strength, which is related to the proportion of variance in the phenotype explained by the genetic variants (R^2^), sample size (n) and number of instruments (k) by the formula F = R^2^ / (1 – R^2^) × (n – k – 1) / k. The proportion of variance in the phenotype explained by each variants is calculated as R^2^ = 2 × β × minor allele frequency × (1 – minor allele frequency).

Supplementary Table S4. Heterogeneity statistics in association estimates and directional pleiotropy detected by the MR Egger regression

| **Genetic score** | **No. of SNPs** | **Heterogeneity Q statistic** | **P value for Q** | ***I*^2^** | **MR Egger intercept** | **Intercept standard error** | **P value for MR Egger intercept test** |
| --- | --- | --- | --- | --- | --- | --- | --- |
| HMGCR score | 4 | 5.807 | 0.121 | 48.3% | -0.148 | 0.148 | 0.316 |
| PCSK9 score | 8 | 9.294 | 0.232 | 24.7% | -0.112 | 0.061 | 0.068 |
| NPC1L1 score | 2 | 0.460 | 0.498 | 0.0% | na**^*^** | na | na |
| LDL score | 67 | 147.382 | **3.7 × 10^-8^** | 55.2% | -0.035 | 0.016 | **0.036** |

**^*^** Not applicable. The MR Egger regression requires data on more than two variants.

Supplementary Table S5. Association of HMGCR genetic score with HLA-B27 positivity and gender

|  | **HLA-B27+**  **(n = 416)** | **HLA-B27-**  **(n = 63)** | **Odds ratio (95% CI) ^*^** | **P value** |
| --- | --- | --- | --- | --- |
| HMGCR score (mean±SD) | 0.1432±0.06199 | 0.1440±0.05816 | 0.97 (0.59-1.60) | 0.919 |
|  | **Male**  **(n = 376)** | **Female**  **(n = 103)** | **Odds ratio (95% CI) ^‡^** | **P value** |
| HMGCR score  (mean±SD) | 0.1443±0.06165 | 0.1432±0.06398 | 1.07 (0.58-1.94) | 0.837 |

**^*^** Odds ratio with 95% CI was estimated for the risk of HLA-B27 positivity per change in HMGCR score equivalent to a 1 mmol/L (38.7 mg/dL) reduction in LDL cholesterol.

**^‡^** Odds ratio with 95% CI was estimated for the risk of male gender per change in HMGCR score equivalent to a 1 mmol/L (38.7 mg/dL) reduction in LDL cholesterol.

Supplementary Table S6. Analyses of association between genetically determined LDL cholesterol levels and risk of ankylosing spondylitis using different estimation models

| **Model** | **Odds ratio (95% CI) ^‡^** | **P value** |
| --- | --- | --- |
| Inverse variance weighted (multiplicative random effects) ^*^ | 0.64 (0.43-0.94) | 0.024 |
| Inverse variance weighted (fixed effects) | 0.64 (0.49-0.83) | 7.13× 10^-4^ |
| Maximum likelihood | 0.63 (0.49-0.83) | 7.09× 10^-4^ |
| MR Egger **^†^** | 0.78 (0.47-1.29) | 0.159 |
| Weighted median | 0.98 (0.64-1.49) | 0.919 |
| MR-PRESSO outlier-corrected | 0.75 (0.54-1.03) | 0.085 |

**^*^** Inverse variance weighted (multiplicative random effects) is the primary estimate for the LDL genetic score, which has been presented here for comparisons.

**^‡^** Odds ratio with 95% CI was estimated for the risk of ankylosing spondylitis per change in HMGCR score equivalent to a 1 mmol/L (38.7 mg/dL) reduction in LDL cholesterol.

**^†^** Effect estimate is the mean of the 1000 bootstrap replications with the 95% CI.

Supplementary Table S7. Comparison of estimates for association with risk of ankylosing spondylitis between the primary cohort and the European population

| **Population** | **Odds ratio (95% CI) ^‡^** | **P value** | **Heterogeneity Q statistic** | **P value for Q** | ***I*^2^** |
| --- | --- | --- | --- | --- | --- |
| **HMGCR genetic score** | | | | | |
| Combined population**^*^** | 0.57 (0.38-0.85) | 5.7 × 10^-3^ | 5.807 | 0.121 | 48.3% |
| European population**^†^** | 0.56 (0.36-0.85) | 7.4 × 10^-3^ | 2.609 | 0.456 | 0.0% |
|  | | | | | |
| **PCSK9 genetic score** | | | | | |
| Combined population**^*^** | 0.89 (0.68-1.16) | 0.378 | 9.294 | 0.232 | 24.7% |
| European population**^†^** | 1.09 (0.82-1.44) | 0.548 | 3.831 | 0.779 | 0.0% |
|  | | | | | |
| **NPC1L1 genetic score** | | | | | |
| Combined population**^*^** | 1.50 (0.39-5.77) | 0.556 | 0.460 | 0.498 | 0.0% |
| European population**^†^** | 2.23 (0.32-15.82) | 0.422 | 0.333 | 0.564 | 0.0% |
|  | | | | | |
| **LDL genetic score** | | | | | |
| Combined population**^*^** | 0.64 (0.43-0.94) | 0.024 | 147.382 | 3.7 × 10^-8^ | 55.2% |
| European population**^†^** | 0.82 (0.61-1.10) | 0.189 | 85.987 | 0.050 | 23.2% |

**^‡^** Odds ratio with 95% CI was estimated for the risk of ankylosing spondylitis per change in HMGCR score equivalent to a 1 mmol/L (38.7 mg/dL) reduction in LDL cholesterol.

**^*^** The combined population was the primary cohort of 33,998 individuals enrolled in IGAS European cohort, IGAS East Asian cohort, Chinese cohort, Turkish cohort and Iranian cohort.

**^†^** European population was extracted from the primary cohort, involving a total of 25,968 individuals of European ancestry.

Supplementary Table S8. Association of genetic variants related to inhibition of drug target with risk of coronary artery disease and type 2 diabetes

| **Drug target** | **Outcome** | **No. of cases** | **No. of controls** | **Odds ratio (95% CI) ^‡^** | **P value** |
| --- | --- | --- | --- | --- | --- |
| HMGCR | Coronary artery disease | 22,233 | 64,762 | 0.58 (0.39-0.85) | 0.006 |
| PCSK9 | Coronary artery disease | 22,233 | 64,762 | 0.53 (0.43-0.66) | 9.2 × 10^-9^ |
| NPC1L1 | Coronary artery disease | 22,233 | 64,762 | 0.56 (0.33-0.94) | 0.028 |
|  | | | | | |
| HMGCR | Type 2 diabetes | 62,892 | 596,424 | 1.30 (1.07-1.59) | 0.008 |
| PCSK9 | Type 2 diabetes | 62,892 | 596,424 | 1.04 (0.86-1.27) | 0.665 |
| NPC1L1 | Type 2 diabetes | 62,892 | 596,424 | 1.30 (0.88-1.93) | 0.193 |

**^‡^** Odds ratio with 95% CI was estimated for the risk of outcome standardised for a 1 mmol/L (38.7 mg/dL) reduction in LDL cholesterol levels.

Supplementary Figure S1. Pattern diagrams illustrating causal inferences in mendelian randomisation and colocalisation

Mendelian randomisation holds the assumption that the randomly inherited genetic variant is strongly and directly associated with the exposure factor and affects the outcome only through its effect on the exposure factor. Thus, the causal effect of allocation to an exposure factor on the outcome can be established via an independent pathway, given the fact that potential confounders tend to be balanced and would not influence the outcome as a result of the process of randomisation at birth. Colocalisation would infer the putative causality between two traits in either direction, if the associations were being driven by the same genetic variant or the same gene region. Colocalisation can be used to assess whether two traits are likely to have a shared etiological link and to test whether the observed association between two traits is caused by genetic confounding from the linkage disequilibrium between two different functional variants.


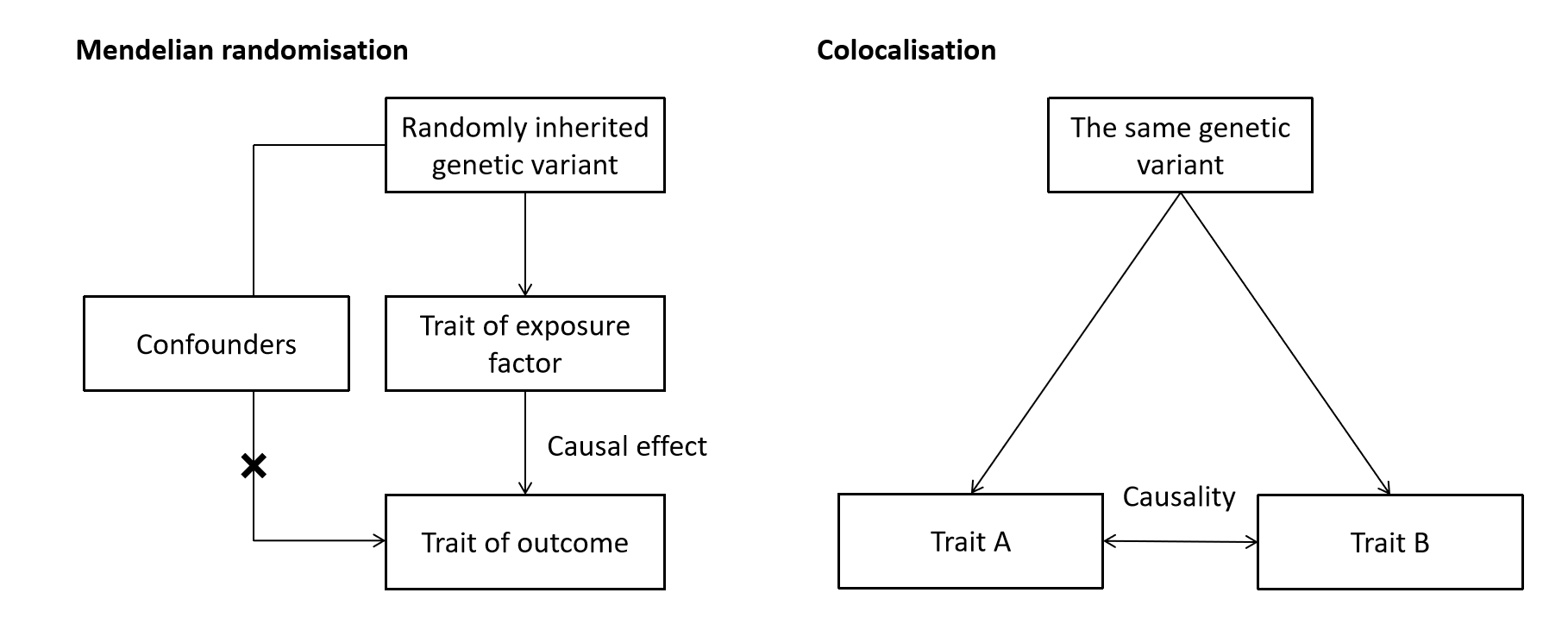


Supplementary Figure S2. Statistical power calculations for primary analyses

Statistical power for each genetic score was estimated to detect an indicated odds ratio (OR) of ankylosing spondylitis (AS) per mmo/L (38.7 mg/dL) change in LDL cholesterol levels at a 5% false positive rate.


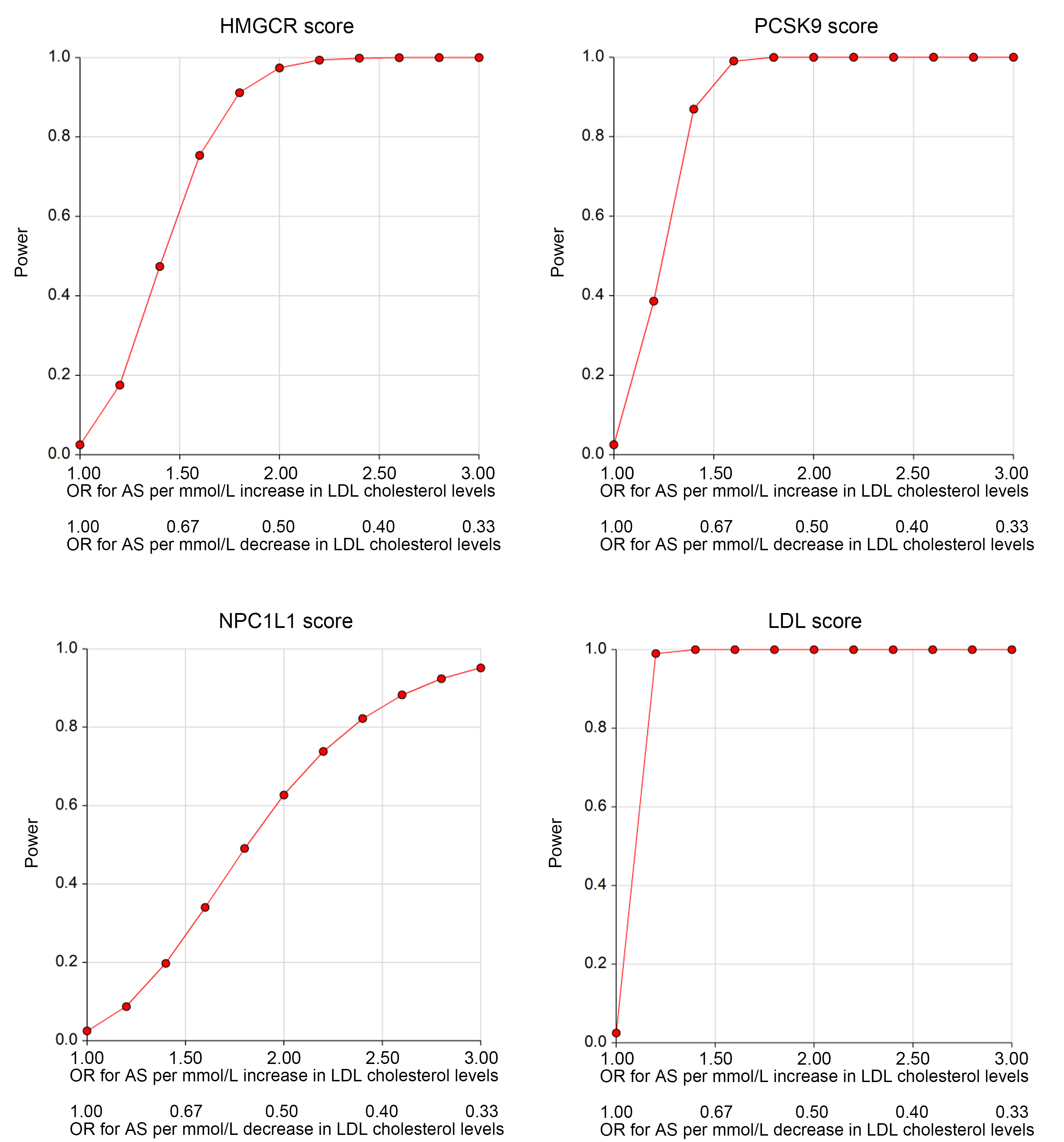


Supplementary Figure S3. Funnel plot of genetic variants included in the LDL genetic score

Effect precision of each genetic variant expressed as the reciprocal of standard error is plotted against its effect size expressed as β coefficient. Vertical lines represent the overall combined effect estimate across variants from inverse variance weighting and MR Egger regression, respectively. Cochrane’s Q statistic was tested to demonstrate the directional pleiotropy. Through visual inspection, data points are largely symmetrically located around the vertical line of the overall combined effect, but some variants with a low precision have a significant deviation, which also indicates the existence of a slight directional pleiotropy.


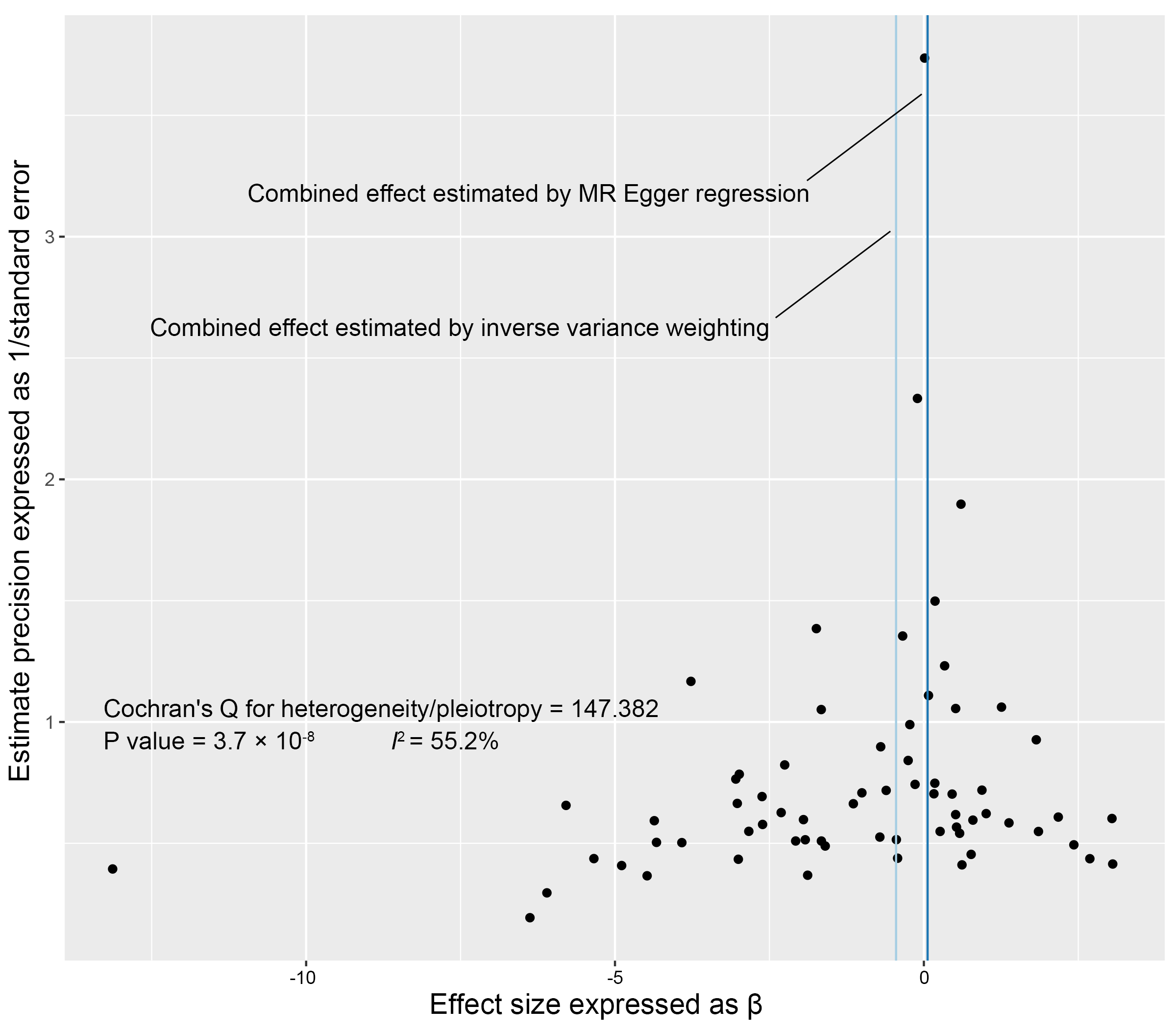


Supplementary Figure S4. Colocalisation analysis of genetic variants in HMGCR

(A) Posterior probability (PPA) of a shared variant between two traits estimated by the Bayesian statistical colocalisation analysis using datasets of the GWAS meta-analysis by the Global Lipids Genetics Consortium and the GWAS meta-analysis of Turkish and Iranian cohorts. Evidence of colocalisation across SNPs proxying HMG-CoA reductase inhibition and ankylosing spondylitis in/near HMGCR has been detected for rs7703051, rs11749783 and rs3846663 with a PPA > 0.01. (B) Linkage disequilibrium (r^2^) matrix showing the perfect pairwise linkage disequilibrium among genetic variants rs12916, rs7703051, rs11749783 and rs3846663, using 1000 Genomes Phase 3 reference panel (CEU Population).

**
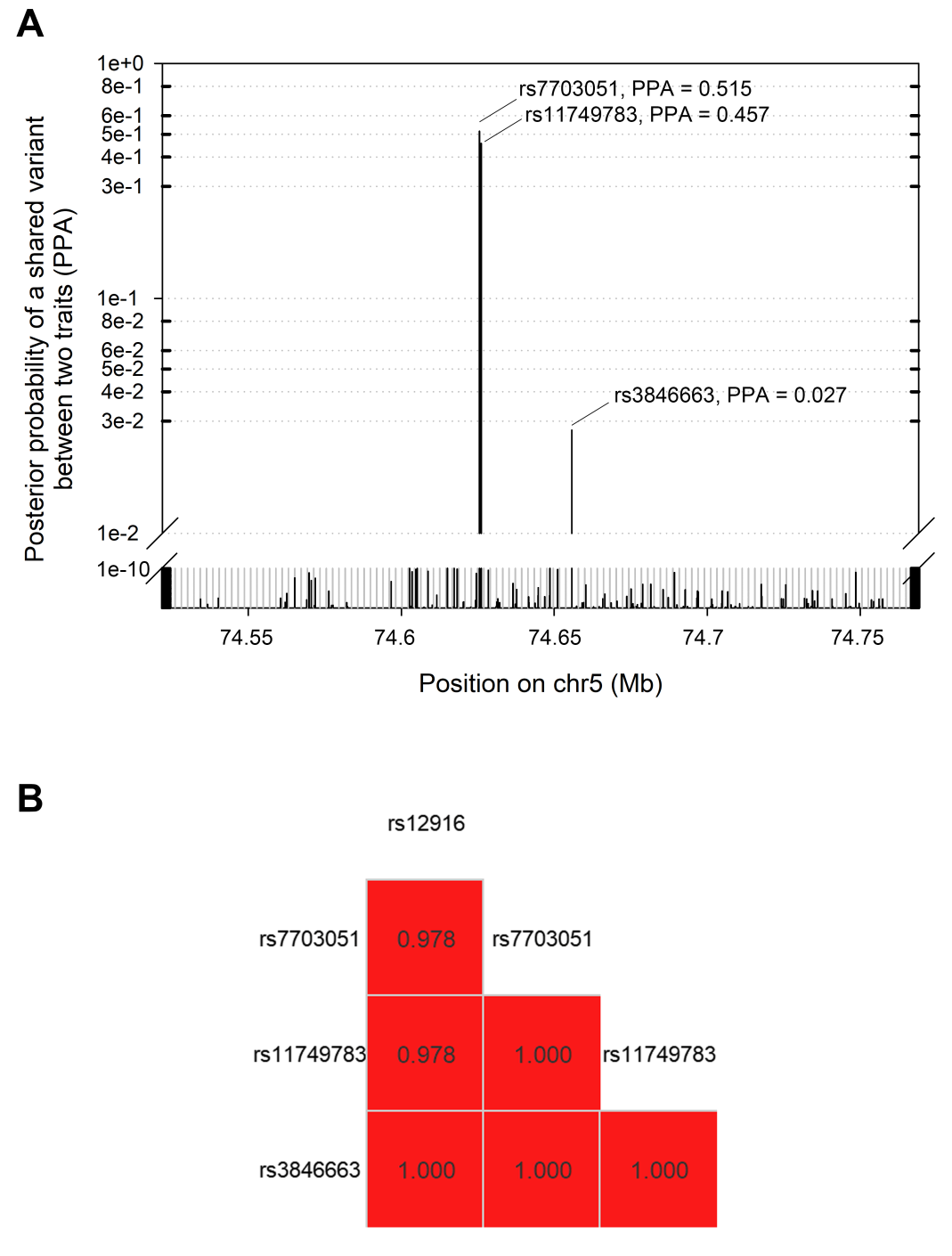
**

References

1. Willer CJ, Schmidt EM, Sengupta S, Peloso GM, Gustafsson S, Kanoni S, et al. Discovery and refinement of loci associated with lipid levels. *Nat Genet* 2013;45(11):1274-83.

2. Burgess S, Butterworth A, Thompson SG. Mendelian randomization analysis with multiple genetic variants using summarized data. *Genet Epidemiol* 2013;37(7):658-65.

3. Burgess S, Dudbridge F, Thompson SG. Combining information on multiple instrumental variables in Mendelian randomization: comparison of allele score and summarized data methods. *Stat Med* 2016;35(11):1880-906.

4. Bowden J, Davey Smith G, Burgess S. Mendelian randomization with invalid instruments: effect estimation and bias detection through Egger regression. *Int J Epidemiol* 2015;44(2):512-25.

5. Bowden J, Davey Smith G, Haycock PC, Burgess S. Consistent Estimation in Mendelian Randomization with Some Invalid Instruments Using a Weighted Median Estimator. *Genet Epidemiol* 2016;40(4):304-14.

6. Verbanck M, Chen C-Y, Neale B, Do R. Detection of widespread horizontal pleiotropy in causal relationships inferred from Mendelian randomization between complex traits and diseases. *Nat Genet* 2018;50(5):693-98.

7. International Genetics of Ankylosing Spondylitis Consortium, Cortes A, Hadler J, Pointon JP, Robinson PC, Karaderi T, et al. Identification of multiple risk variants for ankylosing spondylitis through high-density genotyping of immune-related loci. *Nat Genet* 2013;45(7):730-38.

8. Li Z, Akar S, Yarkan H, Lee SK, Çetin P, Can G, et al. Genome-wide association study in Turkish and Iranian populations identify rare familial Mediterranean fever gene (MEFV) polymorphisms associated with ankylosing spondylitis. *PLoS Genet* 2019;15(4):e1008038.

9. Schunkert H, König IR, Kathiresan S, Reilly MP, Assimes TL, Holm H, et al. Large-scale association analysis identifies 13 new susceptibility loci for coronary artery disease. *Nat Genet* 2011;43(4):333-38.

10. Xue A, Wu Y, Zhu Z, Zhang F, Kemper KE, Zheng Z, et al. Genome-wide association analyses identify 143 risk variants and putative regulatory mechanisms for type 2 diabetes. *Nat Commun* 2018;9(1):2941.

11. Jiang X, O'Reilly PF, Aschard H, Hsu Y-H, Richards JB, Dupuis J, et al. Genome-wide association study in 79,366 European-ancestry individuals informs the genetic architecture of 25-hydroxyvitamin D levels. *Nat Commun* 2018;9(1):260.

12. Matoba N, Akiyama M, Ishigaki K, Kanai M, Takahashi A, Momozawa Y, et al. GWAS of smoking behaviour in 165,436 Japanese people reveals seven new loci and shared genetic architecture. *Nat Hum Behav* 2019;3(5):471-77.

13. Liu M, Jiang Y, Wedow R, Li Y, Brazel DM, Chen F, et al. Association studies of up to 1.2 million individuals yield new insights into the genetic etiology of tobacco and alcohol use. *Nat Genet* 2019;51(2):237-44.

14. Doherty A, Smith-Byrne K, Ferreira T, Holmes MV, Holmes C, Pulit SL, et al. GWAS identifies 14 loci for device-measured physical activity and sleep duration. *Nat Commun* 2018;9(1):5257.
